# ETFT: Equiangular Tight Frame Transformer for Imbalanced Semantic Segmentation

**DOI:** 10.3390/s24216913

**Published:** 2024-10-28

**Authors:** Seonggyun Jeong, Yong Seok Heo

**Affiliations:** 1 Department of Artificial Intelligence, Ajou University, Suwon 16499, Republic of Korea; zkdlghzl5@ajou.ac.kr; 2 Department of Electrical and Computer Engineering, Ajou University, Suwon 16499, Republic of Korea

**Keywords:** semantic segmentation, neural collapse, class imbalance, transformer

## Abstract

Semantic segmentation often suffers from class imbalance, where the label ratio for each class in the dataset is not uniform. Recent studies have addressed the issue of class imbalance in semantic segmentation by leveraging the neural collapse phenomenon in conjunction with an Equiangular Tight Frame (ETF). While the use of ETF aids in enhancing the discriminability of minor classes, class correlation is another crucial factor that must be taken into account. However, managing the balance between class correlation and discrimination through neural collapse remains challenging, as these properties inherently conflict with one another. Moreover, this control is established during the training stage, resulting in a fixed classifier. There is no guarantee that this classifier will consistently perform well with different input images. To address this problem, we propose an Equiangular Tight Frame Transformer (ETFT), a transformer-based model that jointly processes the features and classifier using ETF structure, and dynamically generates the classifier as a function of the input for imbalanced semantic segmentation. Specifically, the classifier initialized with the ETF structure is jointly processed with the input patch tokens during the attention process. As a result, the transformed patch tokens, aided by the ETF structure, achieve discriminability between classes while preserving contextual correlation. The classifier, initially structured as an ETF, is adjusted to incorporate the correlation information, benefiting from the attention mechanism. Furthermore, the learned classifier is combined with the fixed ETF classifier, leveraging the advantages of both. Extensive experiments demonstrate that the proposed method outperforms state-of-the-art methods for imbalanced semantic segmentation on both the ADE20K and Cityscapes datasets.

## 1. Introduction

Semantic segmentation aims at classifying each pixel in an image into a specific class. This task is essential for applications such as road scene analysis for autonomous driving, medical image analysis, and other scenarios where identifying and separating individual objects within an image is crucial [1]. Since the pioneering work of Long et al. [2], neural network-based methods have achieved exceptional performance in this task.

Training semantic segmentation networks usually requires datasets comprising images with their corresponding pixel-wise class annotations. The number of annotations for each class within a single image varies because some major classes occupy larger areas with more pixels compared to minor classes. When aggregating these statistics across the entire dataset, significant disparities in annotation counts for each class may arise. Consequently, semantic segmentation often suffers from a problem known as class imbalance in the training dataset. This issue emerges as a fundamental cause of unequal generalization abilities for each class, causing the trained network to be more significantly influenced by frequently appearing classes. As a result, it causes the features and classifiers of minor classes to be positioned closely, a phenomenon known as neural collapse [3], hindering the network’s ability to discriminate between these classes. In the field of image classification, numerous methods [3,4,5,6,7,8,9,10,11,12,13,14,15] have recently emerged to address class imbalance using the neural collapse phenomenon [3]. This phenomenon occurs in deep learning networks trained on balanced datasets, where both the weights of the last layer acting as classifier and the feature vectors entering this layer exhibit an Equiangular Tight Frame (ETF) structure, meaning the angles between each pair of vectors converge to a maximal equiangular separation. By leveraging this concept, Yang et al. [15] showed that feature learning with a fixed ETF classifier naturally drives the network towards a neural collapse state, even when the dataset is imbalanced across classes.

There have been a few attempts [16,17,18,19,20,21] to solve the class imbalance problem in semantic segmentation. Unlike in image classification, it is more challenging to guide the network toward neural collapse state in semantic segmentation. As Zhong et al. [20] recently explained, the reason is that semantic segmentation easily disrupts the equiangular structure of neural collapse due to inherent contextual correlations between classes and the imbalanced nature of class distributions in the dataset. For this reason, Zhong et al. [20] used the ETF structure solely for regularizing the features, while keeping the classifier learnable. In general, when applying neural collapse to semantic segmentation, it is essential not only to enhance the ability to discriminate minor classes but also to capture contextual correlation information. While the ETF structure can assist in pulling together closely positioned output features for minor classes during training, it may also interfere with the contextualized correlation information learned between classes. Although it is crucial to control the degree of class correlation and discrimination through neural collapse, it is challenging due to the inherently conflicting nature of these properties. Furthermore, this control through neural collapse is achieved during the training stage. However, once the training is complete, the classifier is fixed and there is no assurance that it will consistently adapt well to different input images.

To address this issue, we propose an Equiangular Tight Frame Transformer (ETFT), a transformer-based model that jointly processes the features and classifier using the ETF structure, and dynamically generates the classifier as a function of the input for imbalanced semantic segmentation. Inspired by [20,22], our ETFT can be integrated into the decoders of off-the-shelf transformer-based networks and finetunes them to address the class imbalance problem. Specifically, our ETFT model consists of a sequence of transformer blocks. The first block takes the contextualized patch tokens coming from the decoder of the pre-trained transformer-based networks, along with the additional tokens initialized with the ETF structure for adaptable classifier, as inputs. The last block of ETFT produces both the transformed tokens from the decoder and the adjusted classifier as outputs. In ETFT, the feature and ETF classifier tokens interact globally in the attention process, allowing them to achieve both correlation and discrimination in an adaptive manner. This ensures that the contextualized features adhere to the ETF structure. Meanwhile the classifier, initially structured as an ETF, is trained to encapsulate the entire input information, thereby capturing important context and semantic information that aids in dynamically adapting to the optimal form based on the input feature tokens. As a result, the transformed patch tokens, aided by the ETF structure, achieve discriminability between classes while preserving contextual correlation. The classifier, initially structured as an ETF, is adjusted to incorporate the correlation information, benefiting from the attention mechanism. To further refine the classifier, we employ a simple approach that combines the adapted classifier with the fixed ETF classifier, leveraging the strengths of both. Specifically, our final classifier is derived from the output of additional fully connected layers, which take the concatenation of both classifiers as input.

**Figure 1:**
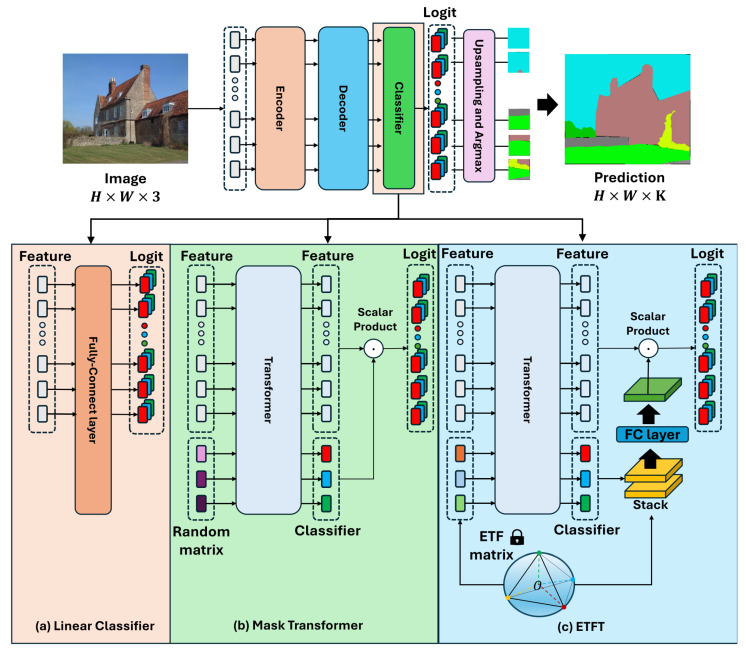
Comparison of classifiers in segmentation networks. (**a**) Linear classifier [20]. (**b**) Mask transformer [22]. (**c**) Proposed ETFT. The lock icon in (**c**) indicates a fixed ETF structure.

Figure 1 compares the processes in which the features from the decoder are fed into the classifier modules, resulting in the generation of logits. The classifier in our ETFT differs from the linear classifier  [20] in Figure 1a. Instead of the static approach of the linear classifier, our model jointly processes feature tokens from the decoder and the learnable classifier. This results in the dynamic generation of a classifier tailored to each input image. Our ETFT also differs from the mask transformer [22] in Figure 1b, in that ETFT initializes the class-embedding vectors with an ETF structure, while the latter generates them from a random matrix, which does not leverage the advantages of the ETF structure.

To demonstrate the superiority of the proposed method, we conducted several experiments applying our approach to various state-of-the-art semantic segmentation networks based on the Transformer [22,23,24,25,26]. Various experiments on ADE20K [27] and Cityscapes [28] show significant improvements of the proposed method over existing methods both quantitatively and qualitatively. The contributions of the proposed method are summarized as follows:

- We propose an Equiangular Tight Frame Transformer (ETFT), a transformer-based model that effectively incorporates the ETF structure within the attention process to simultaneously capture global context for class correlation and enhance discriminability for minority classes in imbalanced semantic segmentation.
- ETFT utilizes the ETF structure to dynamically generate an adaptive classifier for each input image by jointly processing it with the feature tokens from the decoder. Furthermore, we combine a learned classifier with the fixed ETF classifier through fully connected layers, thereby leveraging the strengths of both.
- We demonstrate that our method shows significant improvements in accuracy when seamlessly applied to state-of-the-art transformer-based semantic segmentation networks.

## 2. Related Work

In this section, we provide an overview of approaches related to ours, including transformer-based semantic segmentation and imbalanced semantic segmentation.

### 2.1. Transformer-Based Semantic Segmentation

The vision transformer (ViT) [29] was the first to introduce the transformer architecture, which contains an attention mechanism, for vision tasks. Similar to the development process of CNNs, various transformer-based backbones have been proposed, such as the Swin transformer [30] and the mixed transformer [24].

As transformer-based backbones advanced, the decoders of segmentation networks [22,24,25,26,31,32,33] evolved to adapt to these backbones. Among them, DETR [31] was the first to propose a transformer-based decoder for segmentation tasks. Segformer [24] utilized a lightweight MLP decoder to integrate multi-scale features from the transformer-based encoder. They showed that a lightweight decoder structure can improve performance by mixing multi-scale features. Inspired by DETR, Segmenter [22] proposed a transformer-based decoder called the mask transformer, which uses learnable class-embedding vectors as classifiers. With trained class embeddings and feature vectors, they created a learnable classifier without using layer weights for the classifier. MaskFormer [32] proposed a per-mask classification structure, utilizing a transformer-based decoder and a pixel decoder. Multi-scale features are mixed in the pixel decoder, and the outputs of pixel decoder are passed through the transformer-based decoder with learnable queries to predict mask. Mask2Former [33] was developed from MaskFormer, changing the transformer-based decoder’s attention mechanism from cross-attention to masked attention. SeMask [26] proposed a sub-branch semantic decoder, which also utilizes other segmentation decoders. The semantic decoder contains semantic attention that extracts semantic features. FeedFormer [25] modified the Segformer framework by changing the lightweight MLP decoder to a transformer-based decoder, incorporating cross-attention operations with multi-scale features from the hierarchical encoder. However, the mentioned methods are general semantic segmentation networks that do not consider class imbalance problem. Our proposed method is designed to address the class imbalance problem in semantic segmentation. As such, our proposed method can be easily integrated with existing decoder methods to improve accuracy.

### 2.2. Class-Imbalanced Semantic Segmentation

To address class imbalance in semantic segmentation, various methods [16,17,18,19,20,21] have been proposed. Berman et al. [16] introduced the Lovász–Softmax loss, which optimizes the IoU score for semantic segmentation. This approach better balances tail classes compared to traditional per-pixel loss functions, such as cross-entropy. Tian et al. [17] highlighted that many current methods for tackling class imbalance frequently rely on class distribution to determine weights or margins for the loss function, which may introduce problems. Increasing the weight for tail classes can raise false positives, and tail classes are not always hard cases. To address this, they proposed the recall loss, where the weight of cross-entropy is based on the recall value of each class. DisAlign [19] proposed a two-stage learning approach with adaptive logit adjustment for classifier training. In the first stage, the feature extractor and classifier are jointly trained on an imbalanced dataset. In the second stage, a learnable calibration function is trained on a balanced dataset, while the feature extractor is kept frozen, and then combined with the classifier. PaCo (Parametric Contrastive Learning) [18] imposes constraints on feature vectors by utilizing contrastive loss with a set of parametric learnable class centers, effectively alleviating class imbalance. GPaCo (Generalized Parametric Contrastive Learning) [21] enhances this by adding parametric class-wise learnable centers to regularize the feature space and removing the momentum encoder from the PaCo framework. CeCo (Center Collapse Regularizer) [20] imposes ETF constraints on average class features and jointly trains them with a linear classifier.

## 3. Preliminaries

### Neural Collapse

Papyan et al.  [3] demonstrated a phenomenon called neural collapse, where a classification network trained on a balanced dataset exhibits a Simplex Equiangular Tight Frame (ETF) structure in both the feature vectors entering the final layer and the classifiers at the terminal phase of training. The simplex ETF is defined as a collection of vectors $m_{k}\in R^{d},$ for k=1,2,…,K, where *K* is a number of classes, and d≥K−1, as described in  [3]:

(1) $$M=\sqrt{\frac{K}{K-1}}U\left(I_{K}-\frac{1}{K}1_{K}1_{K}^{T}\right),$$

where $M=\left[m_{1},\ldots ,m_{K}\right]\in R^{d\times K}$, and  $U\in R^{d\times K}$ is a semi-orthogonal matrix which satisfies $U^{T}U=I_{K}$ and allows for a rotation. Here, $I_{K}$ is an identity matrix, and $1_{K}$ is an all-ones vector. All vectors ${\left\{m_{k}\right\}}_{k=1}^{K}$ in a simplex ETF have an equal $l_{2}$ norm and the same pairwise angle, which can be expressed as

(2) $$\begin{array}{c} m_{i}^{T}m_{j}=\frac{K}{K-1}\delta_{i,j}-\frac{1}{K-1},\forall i,j\in \left\{1,\ldots ,K\right\},\\ \delta_{i,j}=\left\{\begin{array}{cc} 1 & \mathrm{if}\;i=j\\ 0 & \mathrm{otherwise}. \end{array}\right. \end{array}$$

The pairwise angle $-\frac{1}{K-1}$ represents the maximal equiangular separation of *K* vectors in $R^{d}$.

Consider a classification network trained in a supervised manner using a dataset composed of *L* training images, denoted as ${\left\{x_{i}\right\}}_{i=1}^{L}$. Let the weight matrix of the network’s final layer, which represent classifiers, be defined as $W=\left[w_{1},\cdots ,w_{K}\right]\in R^{d\times K}$, where $w_{k}\in R^{d}$ represents the classifier corresponding to *k*-th class. The feature vector entering the final layer for $x_{i}$ is denoted as $z_{i}\in R^{d}$. The global mean, $z_{m}$, of the feature vectors for all the *L* images, and the within-class mean, ${\bar{z}}_{k}$, of the feature vectors for *k*-th class are defined as follows, respectively:

(3) $$\begin{array}{c} z_{m}=\underset{i}{Ave}\left\{z_{i}\right\},\\ {\bar{z}}_{k}=\frac{\sum_{i=1}^{n_{k}}z_{i}}{n_{k}}, \end{array}$$

where $n_{k}$ indicates the number of samples in the dataset belonging to *k*-th class. Then, the normalized within-class mean ${\hat{z}}_{k}$ for the *k*-th class can be computed as

(4) $${\hat{z}}_{k}=\frac{{\bar{z}}_{k}-z_{m}}{∥{\bar{z}}_{k}-z_{m}∥}.$$

Similarly, the normalized classifier ${\hat{w}}_{k}$ for the *k*-th class can be computed as

(5) $${\hat{w}}_{k}=\frac{w_{k}}{∥w_{k}∥}.$$

**Figure 2:**
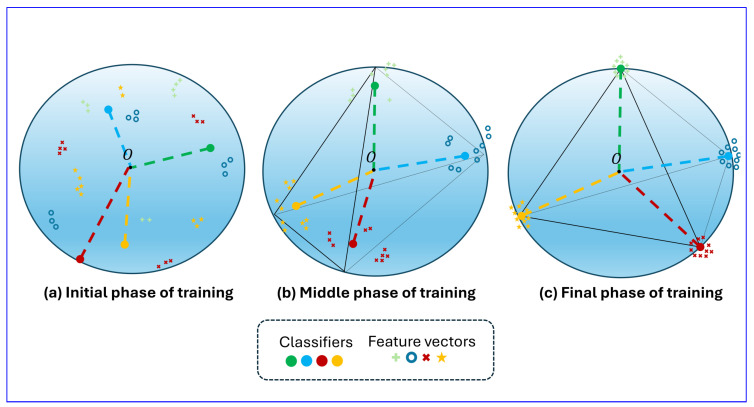
The process of neural collapse in classification networks during training. At the end of training, neural collapse occurs, where both the classifiers (weights of the last layer) and class means of features converge to an Equiangular Tight Frame (ETF) structure. Specifically, they converge to have the same norm, and the angle between any two vectors (except for identical ones) is the same for all pairs.

The neural collapse exhibits following key characteristics  [3]. First, the variance of the within-class feature vectors approaches zero, causing them to cluster around their respective class means. Second, both the normalized within-class mean ${\hat{z}}_{k}$ and the normalized classifier ${\hat{w}}_{k}$ converge to $m_{k}$ in the ETF, satisfying the property in Equation (2). Finally, the classifier converges to selecting the class with the nearest class mean. Figure 2 illustrates the neural collapse process, highlighting the relationship between the feature vectors and classifiers during the training process. At the beginning of training, the classifiers and class means of the features are not aligned. As training progresses, both the classifiers and class means gradually converge towards the ETF structure, with the angles between each pair adjusting to become equal. By the end of training, they fully converge to the ETF structure.

## 4. Proposed Method

**Figure 3:**
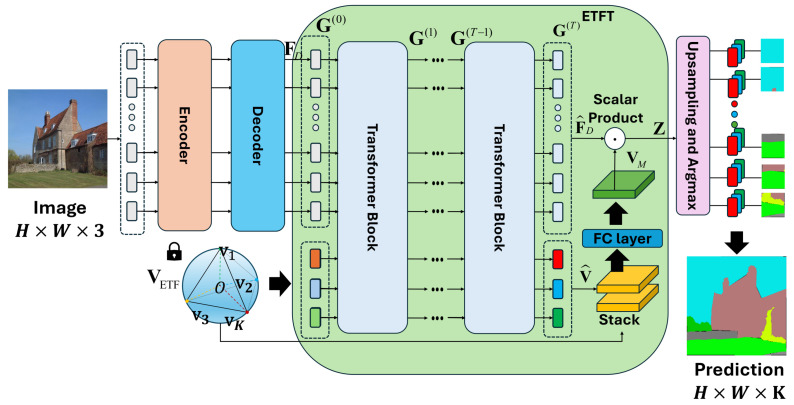
Our proposed module, ETF Transformer (ETFT), is composed of several transformer blocks. The input to the first transformer block is generated by concatenating the feature vectors from the decoder with a fixed ETF matrix. The final classifier is obtained by concatenating the learned classifier with the fixed ETF classifier and passing them through fully connected layers.

Figure 3 shows an overview of the whole segmentation framework including the proposed ETFT structure. In the transformer-based semantic segmentation, the input image is first transformed to a sequence of patches for computational efficiency. Patch embeddings are generated by first flattening the pixel values of each patch and then applying a linear transformation to them. The positional encoding [29] is added to the patch embedding to integrate positional information, resulting in input tokens. The encoder and decoder process the input tokens, generating a sequence of tokens that contain global contextual information. Then, the proposed ETFT is applied to these tokens, along with additional tokens featuring an ETF structure that dynamically changes, generating a set of class embeddings that act as point/pixel-wise classifiers. The output tokens of ETFT are used to perform semantic segmentation tasks as conventional semantic segmentation methods [22,24,25,26,31,32,33,34]. Specifically, the outputs for the patch tokens become a set of logits by performing an inner product between the patch tokens and the class embeddings. Since the resolution of the logits has been reduced, bilinear interpolation is performed to match the resolution of the input image. After that, the logits are converted into a probability distribution through a softmax operation. This distribution for each pixel is used to train the network with a per-pixel cross-entropy loss in an end-to-end manner. During inference, a segmentation map that indicates the class of each pixel is obtained by performing an argmax operation across the class dimension. Our ETFT can be seamlessly integrated into the decoders of existing transformer-based semantic segmentation architectures. As shown in Figure 3, the proposed ETFT consists of a series of blocks, where the output of current block becomes the input to the next block. The first block takes the patch tokens from the decoder and additional tokens initialized with ETF structure as input. The final block produces the transformed patch tokens and learned class masks acting as a learned classifier. This learned classifier is concatenated with the fixed ETF classifier via the fully connected layers to produce the final classifier for point-wise classification. Then, the logit for each patch token is generated by multiplying the transformed patch token with the final classifier. The detailed procedure is described as follows.

### 4.1. Feature Attention Constraint

The ETFT consists of a series of *T* transformer blocks. The first block takes the output of the decoder, $F_{D}\in R^{D\times N}$, along with additional tokens, $V_{ETF}\in R^{D\times K}$, as inputs, where *D* denotes the dimension of the decoder’s output vectors (tokens), *N* represents the number of tokens, and *K* is the number of classes. Here, $V_{ETF}$ is created using predefined *K* class-embedding vectors with a random ETF structure [3] as follows:

(6) $$V_{ETF}=\sqrt{\frac{K}{K-1}}U\left(I_{K}-\frac{1}{K}1_{K}1_{K}^{T}\right),$$

where $U\in R^{D\times K}$ represents a semi-orthogonal matrix and satisfies $U^{T}U=I_{K}$, where $I_{K}$ is the identity matrix, and $1_{K}$ is an all-ones vector. Each row vector in $V_{ETF}$ corresponds to a class embedding vector that has a normalized size of 1 and a maximal angular separation of $-\frac{1}{K-1}$ for pairs of embedding vectors belonging to different classes.

Generally, the *i*-th block in ETFT takes $G^{\left(i\right)}\in R^{D\times (N+K)}$ as input and generates $G^{\left(i+1\right)}\in R^{D\times (N+K)}$ as output, which is then fed into the i+1-th block. The procedure in the *i*-th block is formulated as follows:

(7) $${\tilde{G}}^{\left(i\right)}=MSA\left(LN(G^{\left(i\right)}\right))+G^{\left(i\right)},$$

(8) $$G^{\left(i+1\right)}=FF\left(LN\left({\tilde{G}}^{\left(i\right)}\right)\right)+{\tilde{G}}^{\left(i\right)},$$

where ${\tilde{G}}^{\left(i\right)}$ represents intermediate tokens in the *i*-th block. MSA(·), FF(·), and LN(·), respectively, represent the multi-headed self-attention block, feed-forward layer, layer normalization layer [35]. These block architectures are the same as those of ViT [29].

As stated above, the input to the first block, $G^{\left(0\right)}$, is generated by concatenation of $F_{D}$ and $V_{ETF}$, which is formulated by

(9) $$G^{\left(0\right)}=F_{D}\|V_{ETF},$$

where ‖ denotes the concatenation operation along the channel dimension.

After processing *T* blocks, the outputs of ETFT, $G^{\left(T\right)}\in R^{D\times (N+K)}$, are generated. Here, the first *N* vectors along the channel dimension in $G^{\left(T\right)}$ correspond to the transformed patches tokens from the decoder, denoted as ${\hat{F}}_{D}\in R^{D\times N}$. The last *K* tokens correspond to the classifier $\hat{V}\in R^{D\times K}$ which is adapted to the input image starting from $V_{ETF}$.

### 4.2. Mixed Classifier

To further refine the classifier, we integrate the adapted classifier $\hat{V}$ for class correlation with the fixed ETF classifier $V_{ETF}$ for minor classes, thereby taking advantages of the strengths of both classifiers, in a manner similar to the approach described in  [36]. Our final output classifier $V_{M}$ is formulated as

(10) $$V_{M}=\mathrm{FC}\left(\hat{V}\|V_{ETF}\right),$$

where ‖ represents the concatenation operation and FC(·) denotes two fully connected layers. Now, ${\hat{F}}_{D}$ becomes a sequence of logits $Z\in R^{N\times K}$ by performing an inner product with $V_{M}\in R^{D\times K}$ as following:

(11) $$Z={\hat{F}}_{D}^{T}V_{M}.$$

The subsequent steps involve the same processes as in traditional segmentation methods: rearranging, bilinear interpolation, softmax to obtain the probability map $O\in R^{H\times W\times K}$. The $Z\in R^{N\times K}$ is rearranged to the resolution of h×w×K, where N=hw is the number of tokens from the decoder. Since the resolution of the logit **Z** is reduced from H×W to h×w during patch embedding, where $h=\frac{H}{P}$ and $w=\frac{W}{P}$, with *P* representing the size of each patch, bilinear interpolation is used to upsample the logit back to the original input image resolution of H×W. After that, the logit is converted into probability map **O** through softmax operation. The network is trained end-to-end using a per-pixel cross-entropy loss, optimizing the distribution for each pixel with the AdamW optimizer [37]. During inference, **O** is converted into a final segmentation map $S\in R^{H\times W\times 1}$ using the argmax operation along the class dimension. The entire training procedure is summarized in Algorithm 1.
**Algorithm 1:** Training procedure of ETFT**Input:** $x={\left\{\left.x_{i}\right\}\right.}_{i=1}^{B}\in R^{H\times W\times 3}$: Input image, $y={\left\{\left.y_{i}\right\}\right.}_{i=1}^{B}\in R^{H\times W\times K}$ : Label map

f(·) : Pretrained segmentation network

ϕ : Parameters of f(·), θ : Parameters of ETFT

$N=\frac{HW}{P^{2}}$ : The number of tokens, *P* : The size of patch, *K* : The number of classes

*B* : The batch size, *T* : The number of transformer blocks in ETFT

**Output:**
$O\in R^{H\times W\times K}$: Probability map 1: $V_{ETF}\leftarrow \sqrt{\frac{K}{K-1}}U\left(I_{K}-\frac{1}{K}1_{K}1_{K}^{T}\right)$2:**for** number of training iterations **do**3:   $F_{D}\leftarrow f\left(x\right)$4:   $G^{\left(0\right)}\leftarrow F_{D}\|V_{ETF}$5:   **for** t=0,…,T−1 **do**6:     ${\tilde{G}}^{\left(t\right)}\leftarrow MSA\left(G^{\left(t\right)}\right)+G^{\left(t\right)}$7:     $G^{\left(t+1\right)}\leftarrow FF\left(LN\left({\tilde{G}}^{\left(t\right)}\right)\right)+{\tilde{G}}^{\left(t\right)}$8:   **end for**9:   $G^{\left(T\right)}\in R^{D\times (N+K)}$10:   ${\hat{F}}_{D}\in R^{D\times N}\leftarrow G^{\left(T\right)}\left[:,:N\right]$11:   $\hat{V}\in R^{D\times K}\leftarrow G^{\left(T\right)}\left[:,N:\right]$12:   $V_{M}\leftarrow \mathrm{FC}\left(\hat{V}\|V_{ETF}\right)$13:   $Z\leftarrow {\hat{F}}_{D}^{T}V_{M}$14:   Upsampling **Z**15:   Probability map O← Softmax(**Z**)16:   Calculate loss L←CrossEntropy(O,y)17:   Update ϕ, θ←AdamW(ϕ,θ,L)18:**end for**


It is worth noting that the roles of our additional input tokens with ETF structure in our ETFT are twofold: First, the contextualized tokens from the decoder enhance class discriminability by aligning them with the ETF during attention process. Second, the classifier is dynamically generated based on the ETF structure, adapting to the input image to perform semantic segmentation. In the ETFT, the adaptively learnable classifier tokens interact with the input tokens from the decoder during the attention process. Note that, however, different from Segmenter [22] which starts from random class embedding vectors as input, our ETFT utilizes the class embedding vectors with the ETF structure, which effectively facilitates the neural collapse of the feature vectors.

## 5. Experimental Results

To evaluate the performance of the proposed ETFT, we conducted a series of experiments. This section begins by explaining the dataset, metric and implementation details. Following that, we provide extensive comparisons with previous methods to demonstrate the superiority of the proposed approach. Finally, we present a comprehensive ablation study to highlight the contribution of each component in the proposed ETFT.

### 5.1. Dataset, Metric and Implementation Detail

#### 5.1.1. Dataset

To investigate the effectiveness of ETFT, we conducted experiments on two popular datasets for semantic segmentation: ADE20K [27] and Cityscapes [28].

ADE20K contains images with a wide range of classes, totaling 150, and the resolution of the images varies. The dataset comprises 20,210 images in the training set, 2000 in the validation set, and 3000 in the test set. Each image in ADE20K has a corresponding semantic label map. on the other hand, Cityscapes consists of high-resolution road-scene images with a resolution of 2048×1024. The dataset is composed of 2975 images in the training set, 500 in the validation set, and 1525 in the test set. Each image is paired with its corresponding semantic label map, encompassing 19 classes. Compared to Cityscapes, the inclusion of a larger number of classes in ADE20K results in a more diverse set of scenes, including various types of objects and entities.

For each dataset, classes are categorized into three groups: head, body, and tail, as presented in [19]. Specifically, the grouping is performed based on the ratio defined as follows:

(12) $$\mathrm{Ratio}\;\mathrm{of}\;\mathrm{Class}\;k\;=\;\frac{n_{k}}{\sum_{k=1}^{K}n_{k}},$$

where *K* represents the number of classes in the dataset, and $n_{k}$ represents the number of instances for *k*-th class. If the ratio is greater than 0.01, it falls into the head group; if it is between 0.01 and 0.001, it belongs to the body group; otherwise, it is part of the tail group.

#### 5.1.2. Metric

The accuracy of segmentation is measured using the mean Intersection of Union (mIoU) as an evaluation metric. Here, IoU measures the percentage of overlap between the ground truth and the predicted segmentation map, with values closer to 100% indicating greater similarity to the correct answer. Since IoU is calculated for each class, averaging the IoU values across all classes gives the mIoU. To address the class imbalance issue, improving IoU for classes with relatively fewer pixels, such as the body and tail groups mentioned in the Dataset section, is crucial. Therefore, we focused on enhancing IoU for body and tail classes as well as head class. Consequently, we evaluated the overall mIoU for all classes and separately measured the mIoU for the head, body, and tail classes to compare each model.

#### 5.1.3. Implementation Details

The ETFT can be integrated into the decoder of various transformer-based networks, excluding the final layer (classifier) in the decoder. For evaluations, we conducted experiments using recent transformer-based frameworks including Segformer [24], Segmenter [22], SeMask [26], and FeedFormer [25] as our baseline networks. To implement these approaches, we utilized codes from their official github repository projects. We performed fine-tuning on the backbones and decoder, initializing the parameters with their pre-trained weights. The detailed architecture of the transformer block in Figure 3 is described in Appendix A.

During training, we used the AdamW optimizer [37] with an initial learning rate of $6\times 10^{-5}$ and a weight decay of 0.01. A standard data augmentation pipeline is utilized from mmsegmentation [38]. Specifically, we normalized the image using mean and standard deviation and randomly resized the resolution of image with ratio between 0.5 and 2.0. Subsequently, we randomly cropped the image to a resolution of 512 × 512 for ADE20k and 768 × 768 for the Cityscapes dataset. Finally, a horizontal random flip with a probability of 0.5 is applied. During inference, we resized the smaller edge of the image to the cropped resolution for ADE20K, while preserving the aspect ratio of the input image [25]. For the Cityscapes dataset, we use a sliding window approach with an input size of 768 × 768 [25].

### 5.2. Comparison with Previous Methods

#### 5.2.1. Quantitative Comparison

Comparison with SOTA segmentation networks. Firstly, we compare our proposed method with existing state-of-the-art (SOTA) segmentation frameworks [2,22,24,25,30,32,33,39,40,41,42,43,44,45,46] on the ADE20K and Cityscapes datasets based on mIoU and parameter size. To this end, we integrate the proposed ETFT into the Upernet [23], Segmenter [22], Segformer [24], Feedformer [25], and SeMask [26] frameworks for comparison. As shown in Table 1, the models are categorized into lightweight and advanced models based on their parameter size. Applying our method to lightweight models, including Segmenter-T-Mask/16, Segformer-B0, and FeedFormer-B0, resulted in mean Intersection over Union (mIoU) improvements of 1.73%, 3.65%, and 2.07% on ADE20K, and 0.46%, 2.02%, and 1.09% on Cityscapes, respectively, compared to the original models. In the advanced models, applying our method to Swin UperNet-T, SeMask-T FPN and FeedFormer-B2 resulted in mIoU improvements of 0.81%, 1.63% and 0.86% on ADE20K, and 0.62%, 0.65% and 0.10% on Cityscapes, respectively, compared to existing methods.

Comparison with SOTA imbalanced semantic segmentation methods. To demonstrate the superiority of our ETFT, we compared our method with previous class-imbalanced semantic segmentation methods, including CeCo [20] and GPaCo [21], when applied to UperNet [23], Segformer [24], and FeedFormer [25]. In Table 2, our ETFT demonstrates greater mIoU improvements across the head, body, tail, and all groups compared to other methods on the ADE20K dataset. Specifically, for UperNet with Swin-Tiny, our method achieves mIoU improvements of 1.11%, 1.74%, 0.06%, and 0.81% for the head, body, tail, and all groups, respectively, consistently surpassing the performance of other methods. For Segformer-B0 with MiTB0, the mIoU gains of our method are 2.21%, 3.49%, 4.17%, and 3.65%. For FeedFormer-B0 with MiTB0, ETFT shows mIoU increases of 1.34%, 2.43%, 2.02%, and 2.07% compared to result without our module. ETFT shows better improvements over the baseline compared to CeCo and GPaCo, with increases of 0.06%, 4.17%, and 2.02% in UperNet, Segformer-B0, and FeedFormer-B0, respectively, in the tail group.

It is worth noting that CeCo and GPaCo utilize additional loss functions to enhance the discriminability of features for minor classes, such as body and tail classes. However, focusing exclusively on improving the discriminability of features may lead to sub-optimal results. In contrast, the proposed ETFT’s attention mechanism strikes a balance between discriminability and class correlation, resulting in superior performance compared to CeCo and GPaCo. Furthermore, ETFT can dynamically generate an adaptive classifier for each image during inference, whereas CeCo and GPaCo rely on static classifiers with fixed values. As a result, ETFT can consistently adapt the classifier to each image, leading to improved performance across a variety of images.

#### 5.2.2. Qualitative Comparison

**Figure 4:**
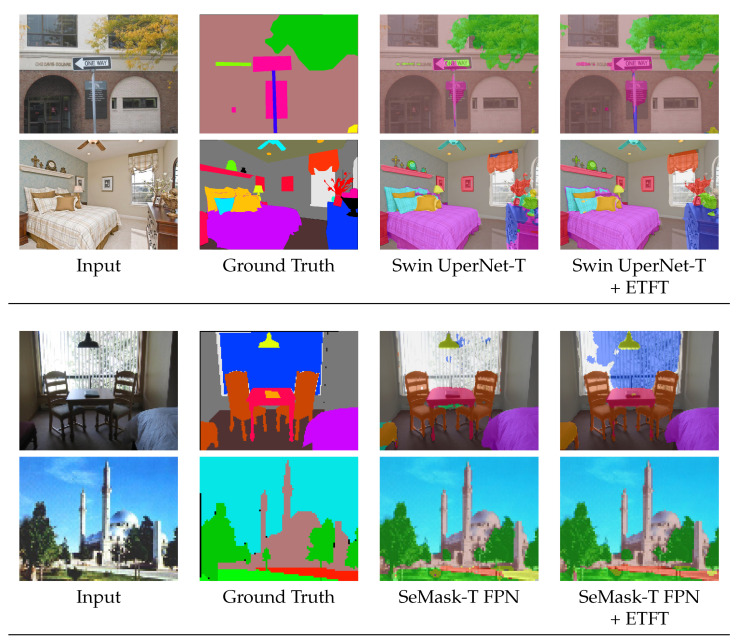

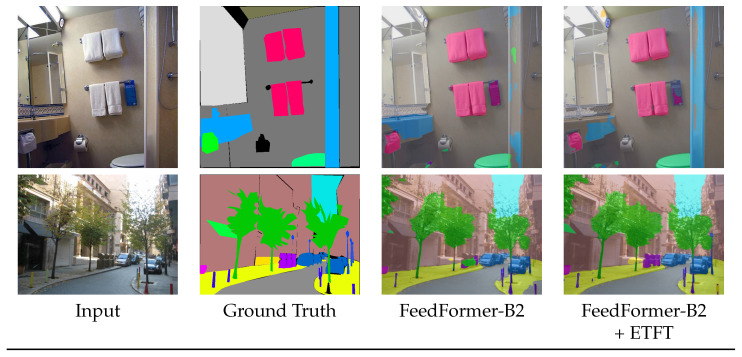
Qualitative comparison of Swin UperNet-T [23], SeMask-T FPN [26], and FeedFormer-B2 [25], without and with our proposed ETFT, on the ADE20K validation set.

**Figure 5:**
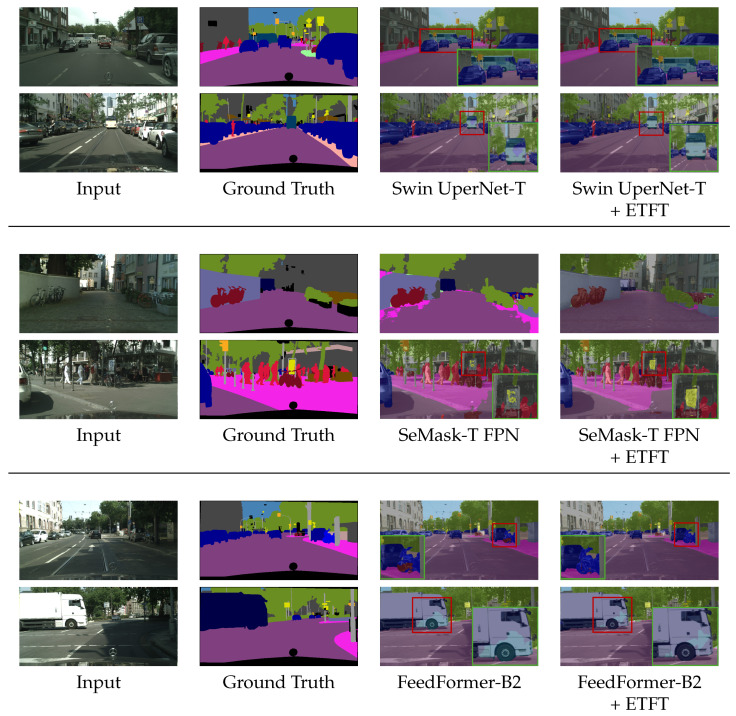
Qualitative comparison of Swin UperNet-T [23], SeMask-T FPN [26], and FeedFormer-B2 [25], without and with our ETFT, on the Cityscapes validation set. The green box represents a magnified view of the corresponding red box.

Figure 4 and Figure 5 show a qualitative comparison of state-of-the-art semantic segmentation networks on ADE20K and Cityscapes validation set, respectively. The results indicate the prediction quality of Swin UperNet-T [23], SeMask-T FPN [26], and FeedFormer-B2 [25], both with and without the proposed ETFT, as shown in Table 1. For ADE20k, in Swin UperNet-T, the traffic sign and drawer parts are mispredicted without ETFT; however, with ETFT, these errors are resolved. Similarly, in SeMask-T FPN, the baseline struggles to accurately capture the blind and the path, but with ETFT, it handles them with ease. Additionally, in FeedFormer-B2, the method with ETFT shows better results in identifying the shower and wastebin parts. Overall, our ETFT enhances the network’s accuracy in predicting scenes involving body and tail classes.

For Cityscapes, we used red and green boxes to highlight the differences. The red box indicates the part we want to highlight, while the green box provides a magnified view of the red box. Focusing on the highlighted areas, in Swin UperNet-T, our module shows more accurate predictions for the small-sized bus class. In SeMask-T FPN, our module results in less noise on the wall and traffic sign. With FeedFormer-B2, our module can better capture small-sized bikes and provides more accurate results for the truck class. As a result, it effectively addresses class imbalance. Our module facilitates more accurate prediction of the tail class compared to before its application.

**Figure 6:**
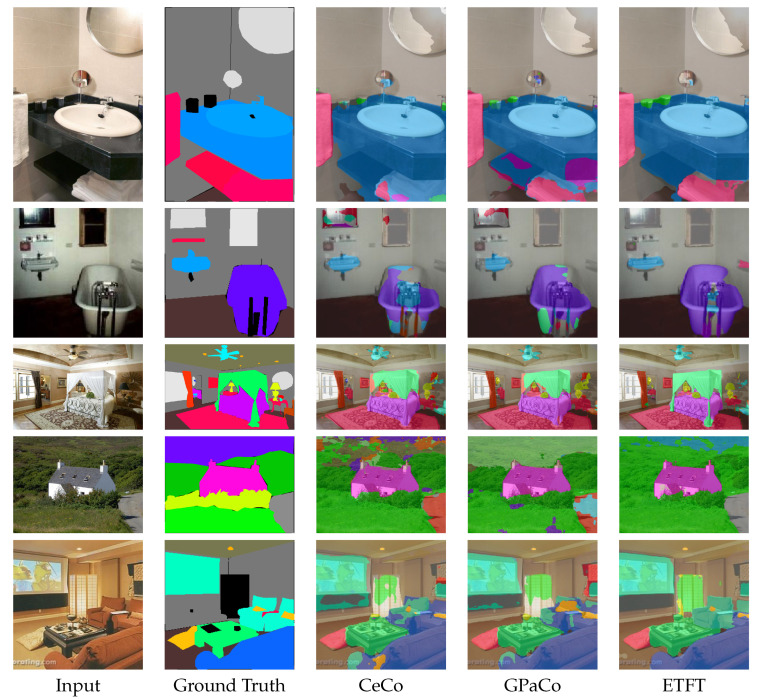
Qualitative comparison of Ceco [20], GPaCo [21] and ETFT on ADE20K validation set.

Figure 6 presents a qualitative comparison with previous class-imbalanced semantic segmentation methods. The baseline framework is FeedFormer-B0 [25], and we applied CeCo [20], GPaCo [21] and our ETFT to this baseline framework to compare the results. The first row shows a comparison in the sink scene, where our method provides better results for the sink and shelf classes. The second and third rows demonstrate that the results using our ETFT are more accurate for the bathtub and covered stand classes, respectively, compared to other methods. Similarly, in the fourth and fifth rows, the road and sofa are classified more accurately with our ETFT compared to previous methods. Overall, the error in the results using our ETFT is significantly reduced, and the predictions are noticeably closer to the ground truth compared to those from CeCo [20] and GPaCo [21] across various scenes.

### 5.3. Ablation Study

Ablation experiments were conducted to verify whether the proposed class embedding, initialized with the ETF structure, and the mixed classifier which combines the learned classifier and the fixed ETF classifier effectively address class imbalance. To this end, four models, including M1, M2, M3, and M4, were trained separately, with all four models sharing the same base framework [22]. Table 3 shows the comparison result of these models.

M1 is identical to the decoder used in Segmenter [22]. The class embedding was initialized randomly and updated as a learnable parameter during training iterations, with the network using the learnable classifier for point-wise classification. For M2, the class embedding was initialized with an ETF structure and then fixed as input during training. The classifier used in M2 was a learnable classifier as M1. The key difference between M2 and M1 is the class-embedding initialization method. Unlike M2, model M3 utilized a fixed classifier with an ETF structure, which was not updated during training. For M4, the class embedding was initialized with an ETF structure as in M2, but combined the learnable classifier with the fixed ETF classifier, forming our proposed mixed classifier. Note that M4 is our proposed model.

The role of the class embedding initialized with the ETF structure is to regulate the updates of the patch tokens from the decoder based on their similarity to the ETF structure, while the mixed classifier aims to enhance the performance of the learnable classifier. As shown in Table 3, M2 shows improvements of 0.15%, 0.59%, 2%, and 1.23% in mIoU for the head, body, tail, and all groups, respectively, over M1. This demonstrates that the proposed ETF class-embedding initialization method is more effective at addressing class imbalance than the previous random initialization method. Comparing M2, which uses the learnable classifier, with M3, which uses the fixed ETF classifier, M3 shows improvements of 0.54% and 0.06% in the body and all groups, respectively. Conversely, M2 outperforms in the tail group by 0.02% and in the head group by 0.27%. This suggests that M2 has strengths in the tail group but shows weaknesses in the body group.

When comparing M4 and M2, M4 shows improvements of 0.12%, 1.15%, 0.06%, and 0.47% in the head, body, tail, and all groups, respectively. When comparing M4 and M3, M4 shows improvements of 0.14%, 0.61%, 0.33%, and 0.41% in the head, body, tail, and all groups, respectively. These improvements indicate that our proposed mixed classifier outperforms using either classifier alone. Moreover, M4 achieves the highest mIoU across all groups, demonstrating that our proposed method effectively addresses class imbalance.

**Figure 7:**
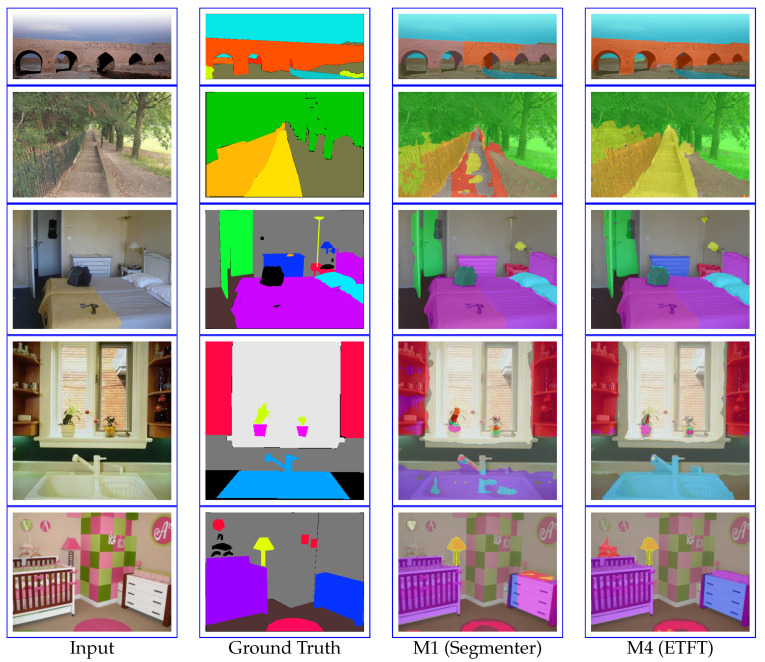
Qualitative comparison of M1 (Segmenter [22]) and M4 (ETFT) on ADE20K validation set.

Figure 7 shows a qualitative comparison between M1 (Segmenter) and M4 (ETFT) on ADE20K. In the first row, M4 provides better prediction results for the bridge compared to M1. In the second row, M1 misclassifies the steps as a path, whereas M2 correctly identifies them as steps. In the third row, M1 misclassifies the drawer, while M4 accurately classifies it with less noise. In the fourth and fifth rows, focusing on the sink and drawer parts, M4 delivers more accurate predictions than M1. For ADE20K, our proposed method demonstrates improved accuracy with reduced noise when predicting previously misclassified classes.

## 6. Limitations

One of the limitations of our method is the increase in the number of parameters. For example, as shown in Table 1, in the case of FeedFormer-B0 [25], the number of parameters increases from 4.43 M to 11.97 M, approximately 2.7 times, when our ETFT is applied to the model. The reason for this increase is as follows: our proposed ETFT retains the encoder–decoder structure of the original segmentation framework, with ETFT attached to the end of the decoder by replacing only the final layer. Since we do not reduce the feature dimension from the decoder in order to preserve it, when the original decoder’s feature dimension is large, the parameters of the query, key, and value embeddings in the transformer block of ETFT increase proportionally.

Another limitation of our method is that the improvement depends on the number of classes in the dataset, with the effect being significant only when the number of classes is sufficiently large. Table 4 shows the differing levels of improvement between ADE20K and Cityscapes. In the case of Segmenter-T-Mask/16 [22], the improvement for all classes is 1.73% in ADE20K, but only 0.46% in Cityscapes. Similarly, for FeedFormer-B0 [25], the improvement of all classes is 2.07% in ADE20K, whereas it is 1.09% in Cityscapes. Overall, the mIoU increase is more significant on ADE20K, which has 150 classes, compared to Cityscapes, which has only 19 classes.

In future work, we believe that there is potential to develop a model that addresses these limitations by achieving effective improvements even in datasets with a small number of classes, while also reducing the number of parameters.

## 7. Conclusions

In this paper, we proposed a ETFT to address class imbalance by incorporating the ETF structure into the transformer architecture for semantic segmentation. By adopting ETFT, the contextualized features and the ETF-based classifier are processed together, with both being dynamically generated based on the input for imbalanced semantic segmentation. To further enhance the classifier, we proposed a straightforward approach that combines the adaptively learned classifier with the fixed ETF classifier, leveraging the strengths of both. Extensive experiments on diverse datasets demonstrate the superior performance of our ETFT compared to recent class-imbalanced semantic segmentation methods, as shown in both quantitative and qualitative evaluations.

## Figures and Tables

**Table 1:** mIoU comparison of semantic segmentation networks on ADE20K and Cityscapes datasets. † indicates our own training results using the official github repository.

	Method	Encoder	Params (M)	ADE20K	Cityscapes
				mIoU	mIoU
Lightweight	FCN [2]	MobileNetV2	9.80	19.70	61.50
	PSPNet [39]	MobileNetV2	13.70	29.60	75.20
	DeepLabV3+ [40]	MobileNetV2	15.40	34.00	75.20
	SwiftNetRN [41]	RseNet18	11.80	-	75.50
	Semantic FPN [42]	ConvMLP-S	12.80	35.80	-
	Segformer-B0 [24] †	MiT-B0	3.77	37.75	73.79
	Segformer-B0 + ETFT (Ours)	MiT-B0	5.75	41.40	75.81
	Segmenter-T-Mask/16 [22] †	ViT-T	6.71	38.22	72.98
	Segmenter-T-Mask/16 + ETFT (Ours)	ViT-T	6.83	39.95	73.44
	FeedFormer-B0 [25] †	MiT-B0	4.43	38.93	76.97
	FeedFormer-B0 + ETFT (Ours)	MiT-B0	11.97	41.00	78.06
Advanced	CCNet [43]	ResNet-101	68.90	43.70	79.50
	DeepLabV3+ [40]	ResNet101	62.70	44.10	80.90
	OCRNet [44]	HRNet-W48	70.50	45.60	81.10
	Auto-DeepLab [45]	Auto-DeepLab-L	44.40	-	80.30
	SenFormer [46]	Swin-T	144.00	46.00	-
	Segmenter-S-Mask/16 [22]	ViT-S	22.00	45.40	-
	MaskFormer [32]	Swin-T	42.00	46.70	-
	Mask2Former [33]	Swin-T	47.00	47.70	82.10
	Swin UperNet-T [23] †	Swin-T	60.00	43.84	79.25
	Swin UperNet-T + ETFT (Ours)	Swin-T	66.44	44.65	79.87
	SeMask-T FPN [26] †	Swin-T	34.24	42.43	77.95
	SeMask-T FPN + ETFT (Ours)	Swin-T	36.46	44.06	78.60
	FeedFormer-B2 [25] †	MiT-B2	29.11	47.19	81.26
	FeedFormer-B2 + ETFT (Ours)	MiT-B2	58.65	48.05	81.36

**Table 2:** mIoU comparison of CeCo [20], GPaCo [21] and our proposed ETFT on ADE20K validation set. The classes are categorized into three groups: head, body, and tail, based on their respective class ratios. Green color indicates an increase in value, while blue color represents a decrease in value compared to the corresponding baseline method.

Method	Encoder	ADE20K			
		Head	Body	Tail	All
UperNet [23]	Swin-Tiny	65.35	45.84	36.63	43.84
+CeCo [20]	Swin-Tiny	66.07 **(+0.72)**	47.12 **(+1.28)**	36.69 **(+0.06)**	44.43 **(+0.59)**
+GPaCo [21]	Swin-Tiny	66.66 **(+1.31)**	47.57 **(+1.73)**	36.59 **(−0.04)**	44.63 **(+0.79)**
+ETFT (Proposed)	Swin-Tiny	66.46 **(+1.11)**	47.58 **(+1.74)**	36.69 **(+0.06)**	44.65 **(+0.81)**
Segformer-B0 [24]	MiT-B0	61.96	40.18	29.51	37.75
+CeCo [20]	MiT-B0	62.39 **(+0.43)**	41.00 **(+0.82)**	30.70 **(+1.19)**	38.70 **(+0.95)**
+GPaCo [21]	MiT-B0	63.61 **(+1.65)**	42.23 **(+2.05)**	30.62 **(+1.11)**	39.27 **(+1.52)**
+ETFT (Proposed)	MiT-B0	64.17 **(+2.21)**	43.67 **(+3.49)**	33.68 **(+4.17)**	41.40 **(+3.65)**
FeedFormer-B0 [25]	MiT-B0	63.06	41.32	30.73	38.93
+CeCo [20]	MiT-B0	62.55 **(−0.51)**	42.49 **(+1.17)**	32.96 **(+2.23)**	40.52 **(+1.59)**
+GPaCo [21]	MiT-B0	64.35 **(+1.29)**	43.11 **(+1.79)**	32.19 **(+1.46)**	40.48 **(+1.55)**
+ETFT (Proposed)	MiT-B0	64.40 **(+1.34)**	43.75 **(+2.43)**	32.75 **(+2.02)**	41.00 **(+2.07)**

**Table 3:** mIoU comparison of ablations for class embedding and classifier in ETFT on the ADE20K validation set.

	M1	M2	M3	M4
Random class embedding	✓			
ETF class embedding		✓	✓	✓
Learned classifier	✓	✓		
Fixed classifier			✓	
Mixed classifier				✓
Head	63.47	63.62	63.60	63.74
Body	42.43	43.02	43.56	44.17
Tail	28.39	30.39	30.12	30.45
All	38.22	39.45	39.51	39.92

**Table 4:** mIoU comparison of Segmenter-T-Mask/16 [22], FeedFormer-B0 [25] and our proposed ETFT on ADE20K and Cityscapes validation sets. The classes are categorized into three groups: head, body, and tail, based on their respective class ratios.

Method	ADE20K				Cityscapes			
	Head	Body	Tail	All	Head	Body	Tail	All
Segmenter-T-Mask/16 [22]	63.47	42.46	28.39	38.22	81.45	66.12	58.42	72.98
Segmenter-T-Mask/16 + ETFT (Ours)	63.74 (+0.27 )	44.17 (+1.71)	30.45 (+2.06)	39.95 (+1.73)	81.57 (+0.12)	66.92 (+0.81)	58.79 (+0.37)	73.44 (+0.46)
FeedFormer-B0 [25]	63.06	41.32	30.73	38.93	84.96	70.38	64.38	76.97
FeedFormer-B0 + ETFT (Ours)	64.40 (+1.34)	43.75 (+2.43)	32.75 (+2.02)	41.00 (+2.07)	85.35 (+0.37)	72.05 (+1.67)	66.44 (+2.06)	78.06 (+1.09)

**Figure A1:**
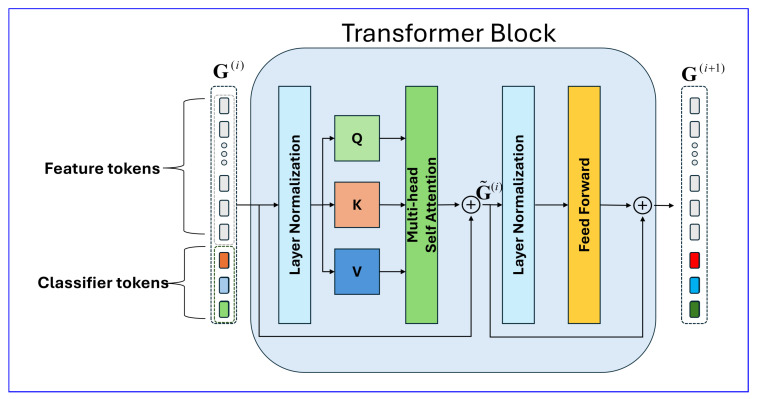
Detailed architecture of a transformer block in ETFT.

## Data Availability

Data are contained within the article. Our code is available at https://github.com/seonggyuny/ETFT.

## Abbreviations

The following abbreviations are used in this manuscript:

ETF	Equinangular Tight Frame
ETFT	Equinangular Tight Frame Transformer
SOTA	State-Of-The-Art
ViT	Vision Transformer
CNN	Convolutional Neural Network
DETR	DEtection TRansformer
FPN	Feature Pyramid Network
PaCo	Parametric Contrastive Learning
GPaCo	Generalized Parametric Contrastive Learning
CeCo	Center Collapse regularizer
mIoU	mean Intersection of Union
IoU	Intersection of Union

## Appendix A. Detailed Architecture of the Transformer Block

As mentioned in the proposed method section, the *i*-th block in ETFT takes $G^{\left(i\right)}\in R^{D\times (N+K)}$ as input and generates $G^{\left(i+1\right)}\in R^{D\times (N+K)}$ as output, which is then fed into the i+1-th block. Figure A1 illustrates the detailed architecture and process of a single transformer block, where the input first passes through a layer normalization layer. Next, it is processed through separate embeddings for query, key, and value, resulting in the creation of the query, key, and value matrices $Q\in R^{D\times (N+K)}$, $K\in R^{D\times (N+K)}$, and $V\in R^{D\times (N+K)}$, which are defined [29] as follows:

(A1) $$\begin{array}{c} Q=\mathrm{Query}\;\mathrm{Embedding}(LN\left(G^{\left(i\right)}\right)),\\ K=\mathrm{Key}\;\mathrm{Embedding}(LN\left(G^{\left(i\right)}\right)),\\ V=\mathrm{Value}\;\mathrm{Embedding}(LN\left(G^{\left(i\right)}\right)), \end{array}$$

where LN(·) denotes the layer normalization [35] operation.

The generated **Q**, **K**, and **V** are used to perform the multi-head self attention [47] operation as follows:

(A2) $$MSA\left(Q,K,V\right)=\mathrm{Softmax}\left(\frac{QK^{T}}{\sqrt{d_{\mathrm{head}}}}\right)V,$$

where $d_{\mathrm{head}}=\frac{D}{N_{\mathrm{head}}}$ is the scaling factor, with a fixed value of 64. The number of heads $N_{\mathrm{head}}$ varies across models to ensure that $d_{\mathrm{head}}$ remains 64. The results of the preceding attention calculation are added to the residual component [29] to obtain ${\tilde{G}}^{\left(i\right)}$, as follows :

(A3) $${\tilde{G}}^{\left(i\right)}=MSA\left(Q,K,V\right)+G^{\left(i\right)}.$$

This ${\tilde{G}}^{\left(i\right)}$ is then passed again through a layer normalization [35] and a feed-forward layer [29], composed of two fully connected layers, after which the residual component is added to generate ${\tilde{G}}^{\left(i+1\right)}$, as follows:

(A4) $$G^{\left(i+1\right)}=FF\left(LN\left({\tilde{G}}^{\left(i\right)}\right)\right)+{\tilde{G}}^{\left(i\right)}.$$

In ETFT, a transformer block that performs the above operation sequentially is repeated, with the default number of blocks set to 2. As shown in Figure A1, the feature tokens and classifier tokens engage in global interaction during the attention process. The classifier tokens, which starts with an ETF structure in the first transformer block, are trained to encompass all input tokens, allowing them to dynamically adapt to their optimal form as classifiers by the final transformer block.
